# Evaluating COVID-19 impact, vaccination, birth registration, and underreporting in a predominantly indigenous population in Chiapas, Mexico

**DOI:** 10.1186/s12879-024-10156-y

**Published:** 2024-12-03

**Authors:** Elienai Joaquin-Damas, Svenn-Erik Mamelund, Benjamin M. Schnneider, Beatriz E. Sánchez-Hernández, Amanda Patishtán-López, Amanda Bleichrodt, Gerardo Chowell

**Affiliations:** 1 Centre for Research On Pandemics & Society (PANSOC), Oslo Metropolitan University, Oslo, Norway; 2 Colegio de Bachilleres de Chiapas No.57, Chiapas, México; 3 Department of Population Health Sciences, School of Public Health, Georgia State University, Atlanta, GA USA

**Keywords:** Indigenous populations, COVID-19, Underreporting, Vaccination, Chiapas, Public health, Health disparities, Health communication, Marginalization

## Abstract

**Background:**

Indigenous populations globally face significant health disparities compared to non-Indigenous groups, primarily due to marginalization and limited access to healthcare. In Mexico, which is home to the largest Indigenous population in the Americas, these disparities were further exacerbated by the COVID-19 pandemic, with impacts intensified by factors such as marginalization, discrimination, and inadequate access to essential services.

**Methods:**

This study aimed to investigate the COVID-19 pandemic's impact on mortality, vaccination access and uptake, and official birth registration among a predominantly Indigenous population in San Juan Chamula, Chiapas. We conducted an online survey among high school students at the Colegio de Bachilleres del Estado de Chiapas, supplemented with epidemiological and socio-demographic data (*N* = 107).

**Results:**

The survey revealed that 14% of respondents reported being infected with COVID-19, while national dashboard data indicated only 212 confirmed cases and one death in Chamula between April 2021 and June 2023. Additionally, 79.4% of respondents were unvaccinated, with significant communication barriers and a lack of information in Indigenous languages contributing to low vaccination rates. Additionally, 5.6% of surveyed family members and 4.7% of community residents lacked official birth certificates, significantly impeding their ability to access essential services such as education, healthcare, and vaccinations.

**Conclusion:**

Our findings highlight significant underreporting of COVID-19 cases and deaths in Indigenous communities, likely due to inadequate diagnostic resources and medical evaluation. The study underscores the urgent need for tailored public health strategies that integrate local Indigenous languages, cultures, and knowledge systems supported by trusted Indigenous leaders. Investing in education in Indigenous languages is crucial for improving vaccination adherence and overall public health outcomes. These strategies can inform national preparedness and response plans to address the unique challenges faced by Indigenous populations during pandemics and other public health crises.

**Supplementary Information:**

The online version contains supplementary material available at 10.1186/s12879-024-10156-y.

## Introduction

Indigenous populations globally often experience poorer health outcomes compared to non-Indigenous groups, largely due to a higher prevalence of comorbidities and limited access to healthcare services [1]. In Mexico, which has the largest Indigenous population in the Americas, these disparities were exacerbated during the COVID-19 pandemic by marginalization, discrimination, and inadequate access to essential services [2, 3].

**Fig. 1:**
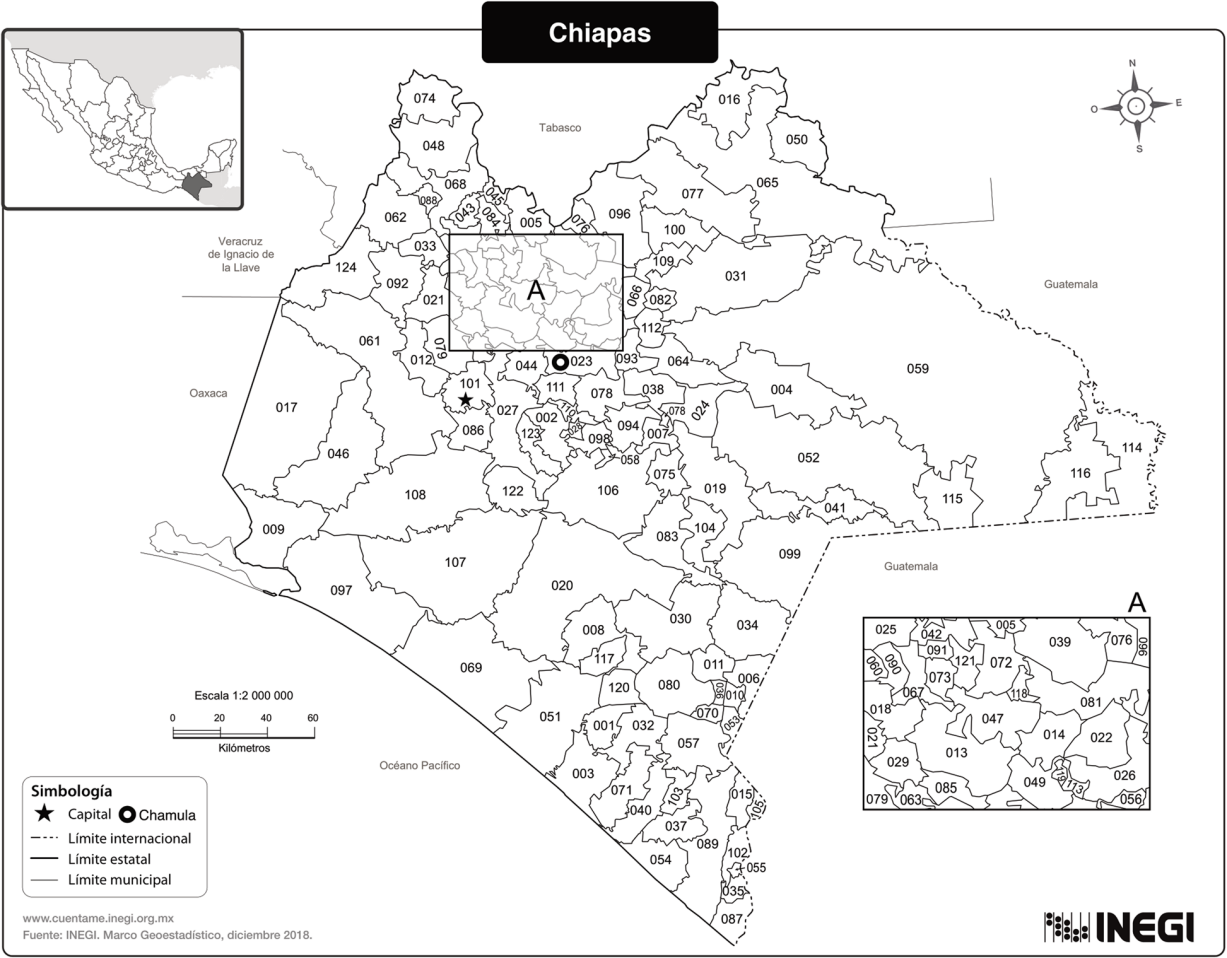
Map of the State of Chiapas showing the 124 municipalities that make up the state. Chamula is the 23rd municipality indicated with a circle [9]

Chiapas, with a total population of 5,543,828 as of March 2020 [2], is one of Mexico’s poorest states and has a considerable Indigenous population (28% of the state’s total population) (see Fig. 1) [2]. The state experiences significant challenges due to restricted access to quality health care, education, and basic infrastructure [3]. These challenges were further magnified during the COVID-19 pandemic, exposing and exacerbating vulnerabilities in the health system and economic structure [4]. A prominent example is the communities in Los Altos Tzotzil-Tzeltal, an area noted for its high population density and marginalization [5], an index that evaluates deficiencies in education, housing quality, income, and socioeconomic conditions [6] in areas where developmental progress is particularly difficult [7]. Spanning 3,723.58 km^2^ and comprising 17 municipalities, Los Altos Tzotzil-Tzeltal is primarily home to the Tsotsil and Tseltal ethnic groups, descendants of the ancient Mayan culture, who continue to maintain their customs and traditions, thereby preserving their culture and identity [8]. The state also faces issues related to education, with a high percentage of children failing to complete primary school.

As of 2020, there were 755,821 inhabitants in Los Altos Tzotzil-Tzeltal [10], and more than 60% of residents (408,958) spoke an Indigenous language [11]. Empirical evidence suggested that COVID-19 was underreported in these regions, according to official COVID-19 records [12]. Motivated by these observations and the region's high degree of marginalization excluding the Municipality of San Cristobal de las Casas, which benefits from significant tourism, we sought to explore and understand the impact of these disparities further. The rest of the municipalities in Los Altos Tzotzil-Tzeltal face the highest percentages of illiteracy in the State, a significant number of children who did not complete primary school, violations of human rights, and a lack of access to essential municipal public services [13]. In the Tsotsil Tseltal region, 15 of the 17 municipalities rank among the 28 with the lowest Human Development Index in the state and among the 100 lowest nationally [11].

According to the National Council for the Evaluation of Social Development Policy (CONEVAL), there is a very high rate of extreme poverty in the Los Altos Tzotzil-Tzeltal region. 43% of the population lack access to nutritious and quality food, 54% face a lack of access to health services because of geographic isolation, high costs, inadequate infrastructure, and shortages of medical professionals. Furthermore, a staggering 95% do not have access to essential services in housing, including clean water, electricity, and proper sanitation [3].

Chamula, situated within the 17 municipalities of Los Altos Tzotzil-Tzeltal, spans a territory of 341 square kilometers [14] and houses 101,967 residents [10]. Predominantly Tsotsil-speaking, Chamula is recognized for preserving elements of its Indigenous identity despite historical attempts by early Spanish colonial efforts to eradicate them in the early sixteenth century [15]. Today, it is celebrated for its touristic tours that showcase ‘the authentic ancestral Indigenous rituals’, illustrating the religious syncretism between the Catholic Christian faith and ancient deity worship [5].

By 2019, the IMSS (Instituto Mexicano del Seguro Social) had a network of 560 small, part-time rural Health clinics throughout Chiapas. In response to COVID-19, in March 2020, the state health department recruited 50 doctors on 6-month contracts to ensure that its primary community hospitals, such as IMSS and SSA (Secretaria de Salud) facilities, remained open [16]. In Chamula, the most used healthcare options in 2020 were the Health Center or Hospital that belonged to the Secretary of Health (SSA), which provides free or low-cost healthcare to uninsured individuals and families [17].

When the mass media reported the WHO’s (World Health Organization) declaration of a pandemic state on March 11, 2020 [18], the majority of the Indigenous population in the area chose not to adhere to the guidelines, including limiting the size and route of religious processions and restricting traditional practices involving close contact and prepared to celebrate “Holy Week” as usual [19], despite the confirmation of the first positive case in Mexico on February 27, 2020. This incident signaled the onset of phase one of the pandemic, attributed to imported contagion. On March 16, 2020, the Chamber of Deputies of Mexico announced the suspension of school activities at all levels [20].

On March 18, 118 confirmed cases of COVID-19 were recorded in Mexico, and on that same day, the Ministry of Health announced the first COVID-19 fatality in the country [21]. By April 30, 2020, every state in the Mexican Republic had only reported at least one case of COVID-19 [21] and Mexico recorded a total of 334,336 deaths by June 2023. Chiapas recorded 59, 903 cases and just 2,454 deaths by June 2023 [22]. However, the municipality of Chamula officially only reported 212 positive cases and one death by June 2023 [23]. Compared to the State capital, Tuxtla Gutiérrez, with 17,650 confirmed cases and 853 deaths [12] there is a low rate of reported deaths in Chamula, indicating an underreporting of this data.

In this study, we collected data that provided a deeper picture of the COVID-19 pandemic in San Juan, Chamula, and Chiapas. Our data document COVID-19 mortality, access to vaccination, and official birth registration in this area with a predominantly Indigenous population in San Juan Chamula Chiapas.

## Methods

This study was designed to investigate the impact of the COVID-19 pandemic on mortality, vaccination access, and official birth registration within a predominantly Indigenous population in San Juan Chamula, Chiapas. To achieve this, we conducted an online survey among high school students at the Colegio de Bachilleres del Estado de Chiapas, which was then supplemented with epidemiological and socio-demographic data. The survey is an extension of the work of Diaz and colleagues [24] and Joaquin-Damas [25], and it included 33 questions implemented via the Google Forms platform (see Supplement 1).

The survey featured a diverse array of question types, including closed-choice, Likert-scale items, multiple-choice, and multiple-select questions, as well as several open-ended questions on vaccination, anthropometric data (weight, height, and Body Mass Index (BMI)), and birth and death registration data. Importantly, participant self-reported weight before the pandemic lockdown (pre-pandemic weight) and their weight two years after the pandemic lockdown (post-pandemic weight), as well as their height. This data was collected to explore potential changes in health indicators, such as BMI, during the pandemic, which could indirectly reflect the impact of COVID-19 on health and lifestyle. This analysis aimed to determine whether these changes were related to pandemic-related restrictions and behaviors, contributing to our understanding of the pandemic's broader health impacts. While this information is important for understanding broader health trends, the primary focus of this study is on the impact of COVID-19 on a marginalized Indigenous population in Chiapas. Therefore, the discussion of BMI is included to provide context.

In collecting data on COVID-19 deaths within the community, we surveyed multiple participants who may have reported the same events. Given that the survey participants were drawn from the same community, double-counting is possible when reporting COVID-19 deaths within the community. While efforts were made to aggregate and cross-reference the data to minimize this risk, some overestimation might have occurred, which is considered a study limitation. As such, considering this potential for double-counting, the results related to the number of COVID-19 deaths in the community should be interpreted with caution.

### Survey’s target population and data collection procedure

We selected high school students as the survey population for several important reasons. First, these students represent an accessible and knowledgeable segment of the community, with the ability to provide insights into their own experiences and the broader context of their families and communities. High school students are often more engaged with both digital platforms and social networks, making them valuable conduits of information within their households**.** Additionally, in many Indigenous communities, youth are frequently tasked with translating and relaying health information to their families due to language barriers and lower literacy rates among older generations. This role places them in a unique position to provide reliable data on community health issues, including the impact of COVID-19.

After high school authorities approved the survey, the Academic Coordinator of Colegio de Bachilleres de Chiapas Plantel San Juan Chamula administered it in the school's computer room using Google Forms. The survey was conducted over one month, from September 15th to October 15th, 2023. Each student received an informed consent form detailing the procedures involved. Students aged 18 and above could participate by voluntarily signing the informed consent form, while those under 18 were required to obtain parental consent. In our study, an individual is considered part of an Indigenous group if they identify as belonging to an autochthonous population or speaking an Indigenous language [26, 27].

While the survey offers valuable insights into the experiences of high school students in San Juan Chamula, it is important to recognize that the data on family members' COVID-19 experiences are based solely on the students' perceptions. This introduces potential biases, such as recall bias, and may affect the accuracy of the reported information. As a result, these findings should be interpreted cautiously, considering the second-hand nature of the data, which may lead to inaccuracies or an overestimation of family members' health experiences.

Our study adhered to strict ethical standards and regulations to ensure the anonymity of participants. Informed consent was obtained in accordance with the Helsinki Declaration [28]. The Institutional Review Committee of the Colegio de Bachilleres Plantel 57, in Chiapas State, approved this study. All respondents' anonymity was carefully maintained throughout the study.

We employed SPSS version 25 to analyze the data. Subjects missing information on height, previous weight, or subsequent weight were excluded (11/107), while all other variables were analyzed for all subjects (*N* = 107).

### Epidemiological and socio-demographic data

We complemented our survey results using government epidemiological data from the COVID-19 pandemic and socio-demographics. These indicators include the marginalization index, defined by the National Population Council (CONAPO), a composite measure evaluating deficiencies in education, housing quality, income, and overall socioeconomic conditions [6]. The marginalization index identifies areas with significant deprivation, expressed as a percentage of the population lacking essential services and resources, helping pinpoint regions needing targeted policy and resource allocation to address these disparities.

For within-state comparison, we also analyzed these indicators across other municipalities in Chiapas [6]. Additionally, we utilized official data from the National Institute of Statistics and Geography (INEGI) to determine population size and territorial extent [10], ensuring accurate contextualization of the COVID-19 infection and death rates reported on the national dashboard [12]. Further details on these factors can be found in Appendix 1.

## Results

We achieved a response rate of 74.3% from a population of 144 enrolled students. In Table 1, a total of 90 participants identified themselves as Indigenous based on self-identification, while an additional 9 participants were classified as Indigenous due to their fluency in an Indigenous language. Given the cultural significance of language in maintaining Indigenous identity, we considered both self-identification and language spoken as critical factors in defining Indigenous status. The participants had an average age of 15 years; the gender distribution was 53.3% male and 46.7% female.

**Table 1:** Demographic characteristics of survey participants (*N* = 107)

Variable	% (*N* = 107)	
Gender	Male 53.3% (*n* = 57)	Female 46.7% (*n* = 50)
Speaks a dialect	Yes 84.1% (*n* = 90)	No 15.9% (*n* = 17)
Considers himself/herself as an Indigenous Person	Yes 84.1% (90)	No 15.9% (*n* = 17)
Indigenous People in this study	92.5% (*n* = 99)	

The average height for these adolescents was 1.57 m (see Table 2), with a notable weight gain of 3.6 kg from pre-pandemic to post-pandemic periods (Wilcoxon test; *P* < 0.001). A significant rise in BMI was also recorded between the pre-pandemic and post-pandemic phases (Wilcoxon test; *P* < 0.001). Since the participants are adolescents, natural growth may have contributed to weight changes. However, the observed increase in weight and BMI might also reflect lifestyle changes during the pandemic, such as reduced physical activity or altered dietary patterns. However, these secondary findings are presented for contextual purposes only.

**Table 2:** Average anthropometric data of survey participants before and after the COVID-19 pandemic (*N* = 96)

Variable	Mean + SD (*N* = 96)	
	Pre-pandemic data	Post-pandemic data
Age	14 to 20 years µ = 15.9 ± 1.09 years	
Height	1.57 ± 0.10 m	
Weight	51.2 ± 21.04 kg	54.8 ± 21.99 kg^a^
BMI	20.07 ± 8.62 kg/m2	22.1 ± 9.05 kg/m2^a^

*SD* Standard Deviation

^a^Wilcoxon signed-rank test statistically significant (*P* < *0.001*)

Concerning COVID-19 outcomes, 14% of participants reported at least one infection during the pandemic (see Table 3), and 38.3% of participants indicated that a family member at home (primarily parents) contracted COVID-19. However, access to COVID-19 testing was rather limited because the cost of a PCR test for COVID-19 was 17 to 35 times the minimum wage (6 to 7 USD per day) in 2021 in Chiapas State. By mid-2024, the minimum wage had increased to 12 to 13 USD per day, and the PCR test for COVID-19 was equivalent to 3 to 6 minimum wages [29, 30]. The predominant treatment among respondents was traditional, with 15.9% utilizing rituals by shamans or healers. Only 7.4% opted for biomedical treatment (see Table 4). Nevertheless, participants might have used more than one treatment option simultaneously.

**Table 3:** COVID-19 illness and outcomes among survey participants and their relatives (*N* = 107)

Variable	% (n)	
	**Yes**	**No**
Subjects who got sick from COVID-19	14% (15)	86% (86)
Relatives of respondents who got sick from COVID-19	38.3% (41)	61.7% (66)
Relatives of respondents who were hospitalized due to COVID -19	6.5% (7)	93.5 (100)
Relatives of respondents who died due to COVID -19	4.7% (5)	95.3% (102)

**Table 4:** Types of treatment received by survey participants during COVID-19 illness (*N* = 107)

Treatment of respondents attended during the illness (COVID -19)	% (n)
Traditional medicine of my community (rituals, shaman, healer)	15.9% (17)
Medicine in a public hospital	2.8% (3)
Medicine in a private hospital	0.9% (1)
Medicine at Dr. Simi's pharmacy, Farmacia del Ahorro or equivalent	3.7% (4)
None of the above	2.8% (3)

COVID-19 fatalities occurred in the homes of 4.7% (see Table 3) of the survey participants. Additionally, 32.7% of the respondents reported the occurrence of COVID-19 deaths in their community (see Table 5), with the number of deaths reported per incident ranging from one to five**.** Notably, 17.8% of these deaths were not officially registered (see Table 6).

**Table 5:** Reported COVID-19 deaths in the community among survey participants (*N* = 107)

Deaths due COVID-19 in the community % (n)		Number of deaths (*N* = 107) % (n)				
Yes	No	1 a 5	6 a 10	11 a 15	> 20	Do not know
32.7%	28%	18.7%	17.8%	2.8%	3.7%	48.6%
(*n* = 35)	(*n* = 30)	(*n* = 20)	(*n* = 19)	(*n* = 3)	(*n* = 4)	(*n* = 52)

**Table 6:** Registration of COVID-19 deaths as reported by survey participants (*N* = 107)

Deaths due to COVID-19 registered	% (*N* = 107)
In the committee of the community	4.7% (5)
In the registry of the church of the community	0.9% (1)
At the municipality	2.8% (3)
Not registered	17.8% (19)
Do not know	73.8% (79)

**Fig. 2:**
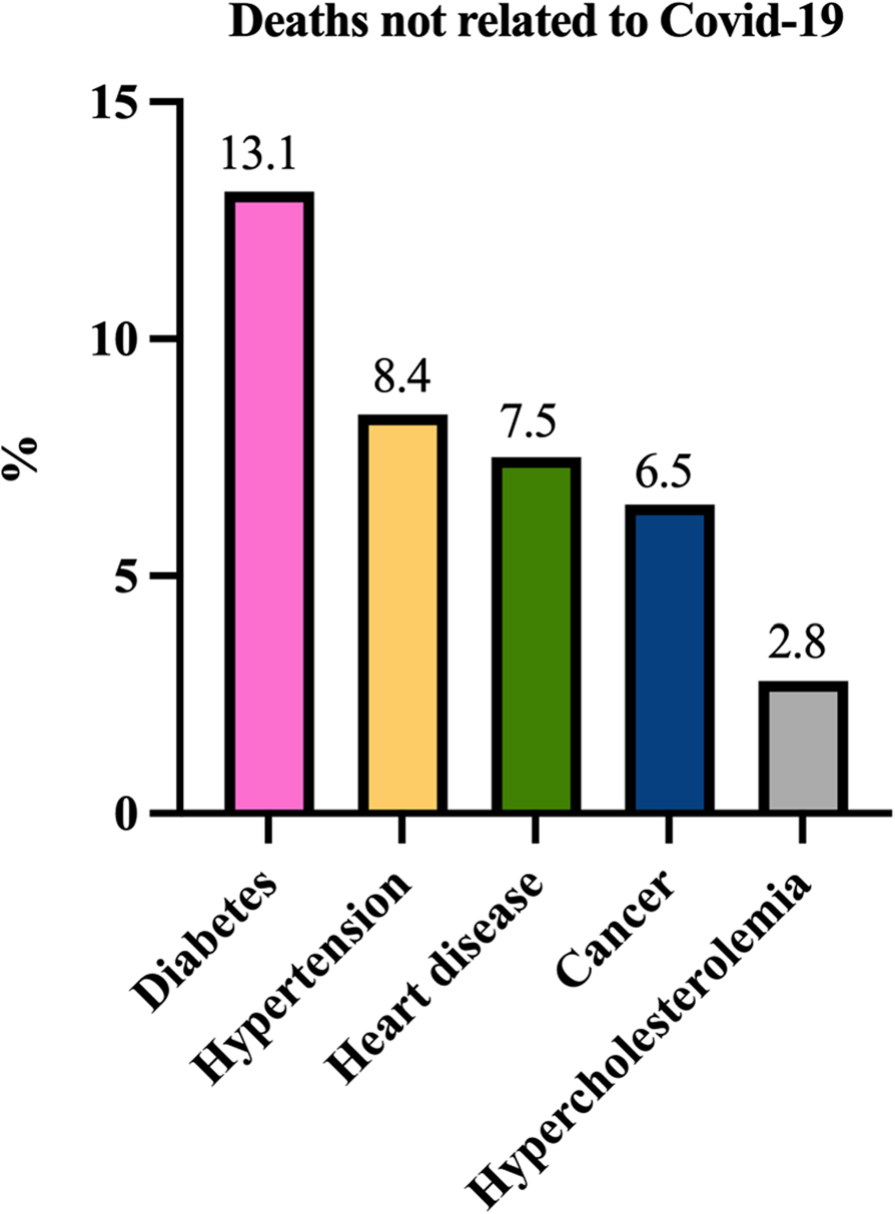
Distribution of other deaths not related to COVID-19 in their community

As for deaths unrelated to COVID-19, the most common death causes reported by the respondents were diabetes, followed by hypertension and cardiovascular diseases (see Fig. 2), aligning with the leading causes of excess mortality during the COVID-19 pandemic in Mexico [31].

Regarding COVID-19 vaccination, more than half of the respondents reported not being offered a vaccine. Indeed, 79.4% were unvaccinated at the time of the survey (see Table 7). The vaccination program in Chiapas was administered primarily by the state health department, with support from federal health authorities. Vaccination was free of charge, ensuring accessibility for all residents, including those in Indigenous communities. Efforts were made to reach Indigenous communities by setting up mobile vaccination units and conducting outreach programs in remote areas. Additionally, information campaigns in Indigenous languages were launched to ensure that the communities were well-informed about the benefits and availability of the vaccines.

**Table 7:** COVID-19 vaccination status and experiences among survey participants (*N* = 107)

You have been offered the vaccine % (n)		Vaccinated against COVID-19 % (n)	
Yes 47.7% (51)	No 52.3% (56)	Yes 20.6% (22)	No 79.4% (85)
**Doses of vaccines administered** **% (n)**			
None 80.4% (86)	1 10.3% (11)	2 7.5% (8)	3 0.9% (1)
**The place where you were vaccinated** **% (n)**			
At the clinic in the community 7.5% (8)	Vaccination campaign in the community 3.7% (4)	Vaccination campaign at school 4.7% (5)	In the nearest city 2.8% (3)
**Explanation of the vaccine in native dialect** **% (n)**			
Yes 29% (31)	No 48.6% (52)	Only in Spanish 15.9% (17)	No explanation 6.5% (7)

Among the vaccinated, 7.5% received their shots at a community clinic or health center. Indigenous communities in Mexico were informed about the benefits, safety, and process of COVID-19 vaccination, including where and how to get vaccinated [32]. However, communication barriers persisted: only 29% were informed about the vaccine in their native dialect, 48.6% lacked translated instructions, and 6.5% received no explanation.

Among the respondents’ relatives, 33.6% had not received a COVID-19 vaccine. Of those vaccinated, 30.8% were immunized at their community clinic. However, 36.4% did not receive instructions or explanations in their dialect (see Table 8).

**Table 8:** COVID-19 vaccination status and experiences among relatives of survey participants (*N* = 107)

Relatives of the respondents vaccinated % (n)		The place where the relatives were vaccinated % (n)		Explanation of the vaccine in native dialect to the relatives % (n)	
Parents	27.1% (29)	At the clinic in the community	30.8% (33)	Yes	36.4% (39)
Person over 18 y	6.5% (7)	Vaccination campaign in the community	8.4% (9)	No	36.4% (39)
Person under 18 y	2.8% (3)	Vaccination campaign at school	4.7% (5)	Only in Spanish	13.1% (14)
Grandparents	3.7% (4)	In the nearest city	13.1% (14)	No explanation	3.7% (4)
Without vaccination	33.6% (36)	Without vaccination	43% (46)	Without vaccination	10.3% (11)

**Fig. 3:**
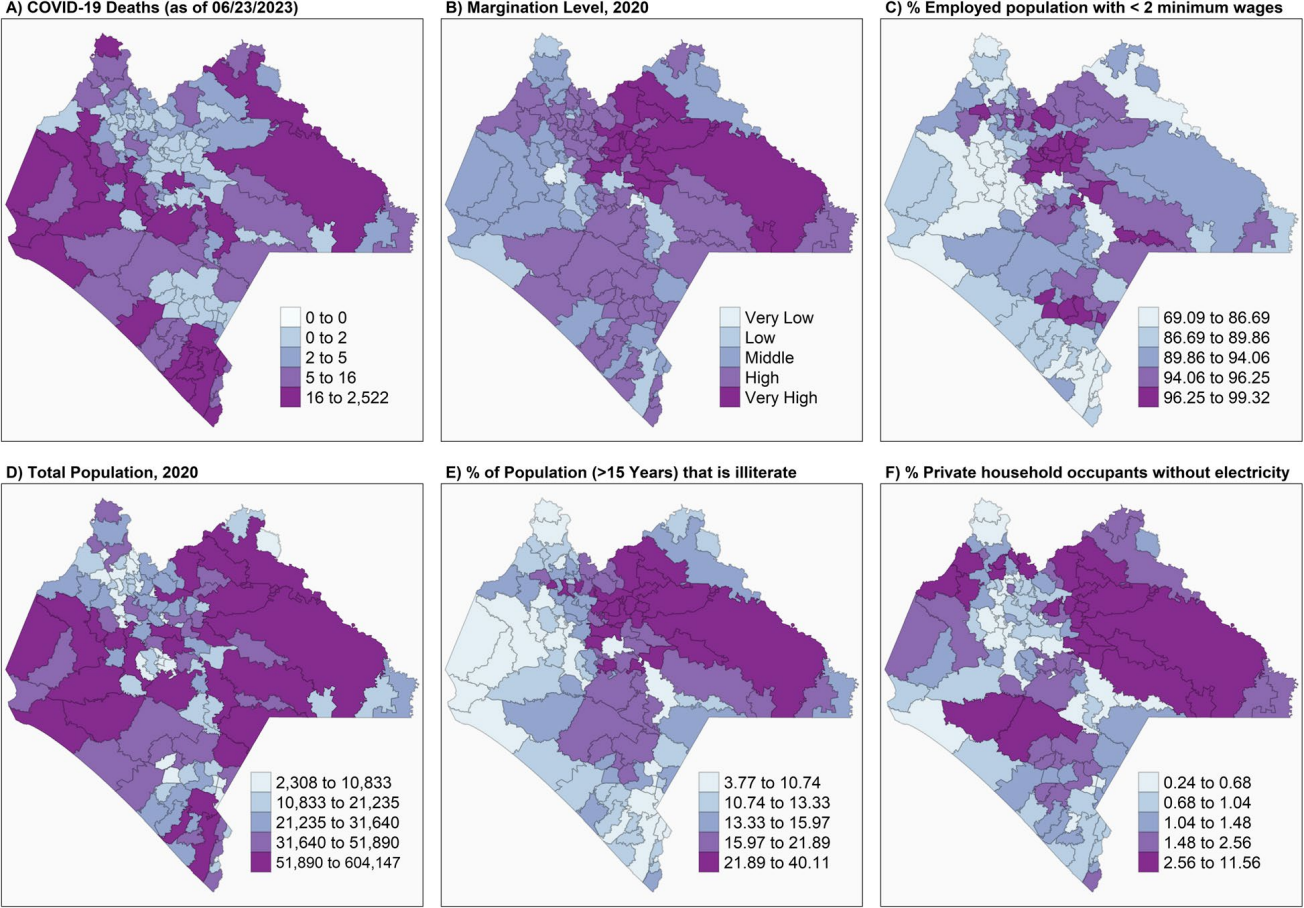
Spatial distribution of COVID-19 deaths and its comparison with % employment, illiterate population, and inhabited houses without electricity in Chiapas State

Figure 3 shows the special distribution of COVID-19 deaths and compares it with the percentages of employment, the illiterate population, and the houses without electricity in Chiapas State.

In reviewing San Juan Chamula’s official data, it can be inferred that other municipalities sharing similar features may also experience similar underreporting issues. Within Chiapas, 20 of its 124 municipalities fall into the Very High Level of Marginalization category (Appendix 2 and 3), accounting for 18.82% of the state's total population. Ocosingo, notable for its vast territorial spread, covers 12.93% of the state’s land and harbors 4.2% of its population, making it the most populated within the highly marginalized municipalities. Yet, it reported only 29 deaths by June 2023. In this way, municipalities experiencing High Marginalization reported between zero and nine deaths during the same period, while those with lower levels of marginalization have more comprehensive data records (Appendix 4).

**Fig. 4:**
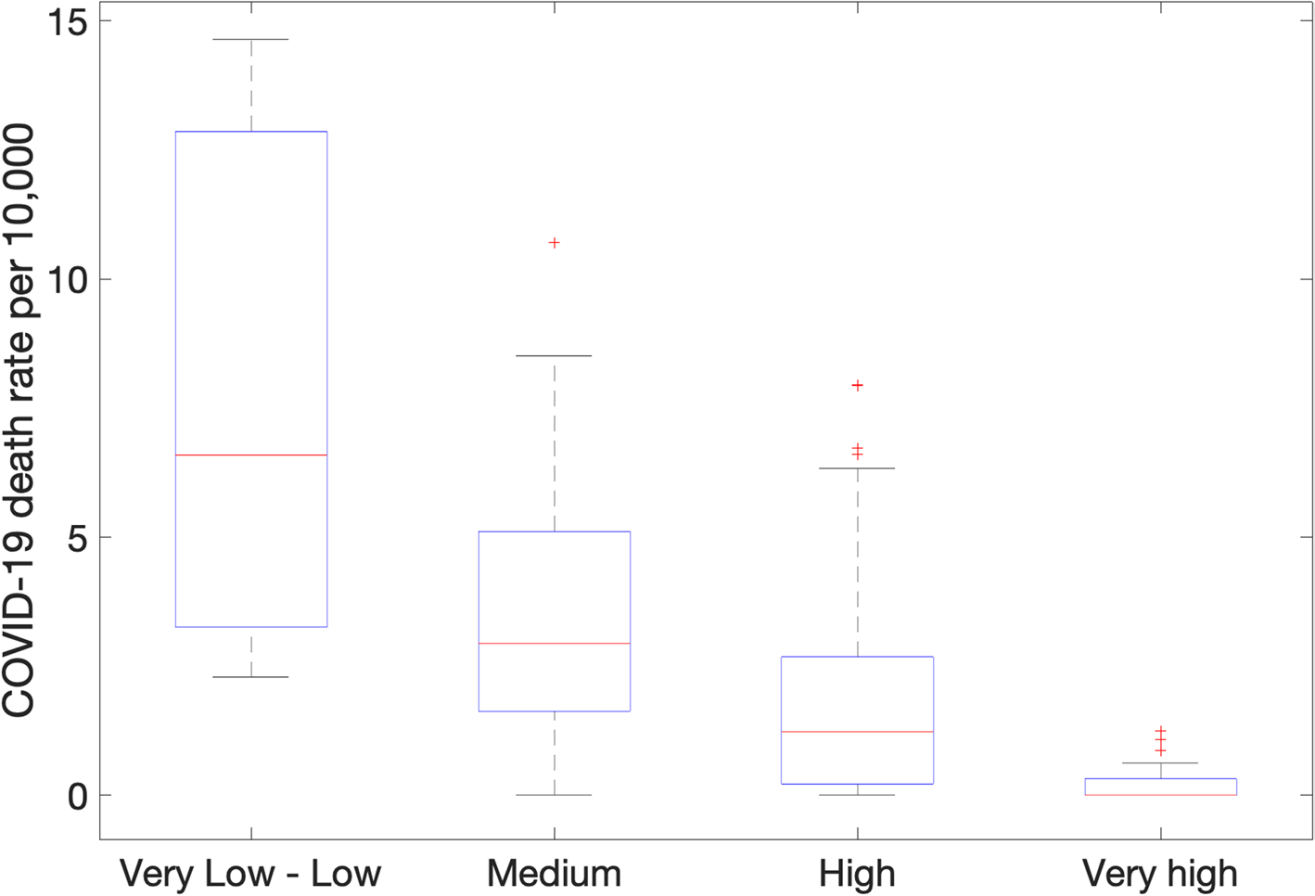
COVID-19 mortality rate per 10,000 inhabitants in the Municipalities of Chiapas by degree of marginalization

Figure 4 depicts the COVID-19 deaths per 10,000 inhabitants in Chiapas’s municipalities, classified by CONAPOS’s marginalization levels. Chiapas’s capital, Tuxtla Gutierrez, is the sole municipality in the Very Low Marginalization category, with a COVID-19 death rate of 14.12. Municipalities with low marginalization exhibit an average COVID-19 death rate of 6.89, while those with very high marginalization record a significantly lower rate of only 0.25. Tuxtla Gutierrez recorded the most deaths (853), in stark contrast to Cholula, which reported only one COVID-19 death.

Notably, birth registration, which is crucial for securing legal identity and accessing various social services, is deficient in the surveyed area. Our findings reveal that 5.6% of surveyed family members and 4.7% of community residents are without an official birth certificate (see Table 9)**.** This lack of documentation impedes individuals' ability to engage fully in societal functions, including voting, enrolling in education, and accessing health care benefits.

**Table 9:** Official birth certificate status among relatives of survey participants (*N* = 107)

Relatives of the respondents have an official birth certificate % (n)		Relatives of the respondents without official birth certificate % (n)	
Yes 94.4% (101)	No 5.6% (6)	Person over 18 y	0.9% (1)
**Somebody else without an official birth certificate in the community** **% (n)**		Person under 18 y	1.9% (2)
Yes	4.7% (5)	Grandparents	0.9% (1)
No	47.7% (51)	Others	13.1% (14)
Do not know	47.7% (51)	Everybody has an official birth certificate	83.2% (89)

## Discussion

Our study highlights significant underreporting of COVID-19 cases and deaths, low vaccination rates, and a notable percentage of individuals without official birth certificates in a predominantly Indigenous population in San Juan Chamula. We find that 79.4% of respondents were unvaccinated at the time of the survey, and 5.6% of family members lacked official birth certificates.

The low reported infection rates, with over 80% of the population group claiming to have remained uninfected during the pandemic, may reflect the geographic isolation of these communities rather than effective containment strategies [33]. This isolation and early community access restrictions likely contributed to the delayed spread of COVID-19. Additionally, the limited availability of public transport during the first wave further reduced the potential for the virus to penetrate these remote areas [34].

According to the respondents, 14% reported being infected with COVID-19 at least once during the pandemic; however, official records in Chamula report only one death attributed to COVID-19 [24]. This discrepancy may suggest underreporting rather than effective community strategies or successful prevention efforts. Gomes-Machado and colleagues highlighted that underreporting among Indigenous populations in Brazil could result from limited diagnostic resources and inadequate medical evaluation [35]. Similarly, in Chamula, it is possible that some COVID-19-related fatalities went undiagnosed and unrecorded, contributing to the significant underestimation of the true mortality burden.

Our findings also reveal that 32.7% of deaths occurred outside family homes, and 17.8% of these deaths were unregistered in any database. These findings underscore the health inequities prevalent in Indigenous communities, driven by social determinants such as marginalization, racism, and precarious living conditions [36]. Disparities in sanitation between Indigenous and urban areas contribute significantly to higher mortality rates in these populations [37]. Additionally, low income and literacy levels further exacerbate health risks, particularly for women [38]. The inadequate treatment of Indigenous people by authorities in Chiapas is exemplified by an incident in April 2020, where police assaulted four Indigenous individuals seeking medical attention during a COVID-19 outbreak. A strict sanitary blockade with a curfew decreed by the authorities prevented them from accessing hospital care, highlighting the systemic suppression of fundamental rights, including access to healthcare, in this region [39].

The use of traditional knowledge by 15.9% of the surveyed population underscores the strong cultural roots of Indigenous communities, contrasting with the 7.4% who relied on Western medicine. These rituals provided physical recovery and emotional, spiritual, and mental resilience during the pandemic [40, 41]. To protect themselves, many communities are isolated by closing off entrances and returning to ancestral practices like hunting, fishing, and using traditional herbs for treatment [42]. Community elders, revered for their vast knowledge of traditional remedies, served as principal healers and were often the only source of healthcare, fostering positive attitudes among residents [40–43]. Despite these efforts, Indigenous populations faced the highest mortality rates during the early waves of COVID-19 [44].

Diabetes emerged as a significant comorbidity in this region, affecting 13.1% of the population as shown in Fig. 2. Globally, approximately 422 million people live with diabetes, with 1.5 million deaths annually attributed to the disease, particularly in low-income countries [45]. Diabetes is a major risk factor for COVID-19 mortality, with Indigenous descent ranking third [46]. In Mexico, chronic conditions such as hypertension and obesity are also strongly associated with COVID-19 mortality, similar to ethnicity [47]. High blood glucose levels are linked to poor outcomes, as diabetic patients have a higher density of angiotensin-converting enzyme-2 receptors in the lungs and pancreas, which SARS-CoV-2 proteins exploit, worsening disease progression [48]. The pandemic exacerbated glucose control issues among diabetic patients due to restricted access to medication, limited medical check-ups, and insufficient self-care, increasing their risk of severe COVID-19 outcomes [49]. Ethnicity further compounds vulnerability, with insufficient knowledge of chronic disease management and medication shortages in communities contributing to higher mortality rates among these populations [37, 47, 50].

The vaccination rate among respondents was notably low, with only 20.6% having received at least one dose and just 27.1% of their parents vaccinated. This contrasts sharply with national data, which shows that 79.8% of the eligible population had received at least one dose [51, 52]. Interestingly, the vaccination coverage among respondents' relatives was significantly higher than among the students. This discrepancy could be due to the prioritization of older or more vulnerable groups during the vaccination rollout[53], as well as logistical challenges in reaching younger individuals or addressing their specific concerns and exposure to misinformation [54, 55].

Language barriers and discrimination further hinder vaccination efforts [56]. The absence of accurate translations and Indigenous communities' unfamiliarity with health systems and technology often result in unsuccessful registration in the national immunization system [57]. For instance, in Australia, many Aboriginal people prefer to be vaccinated by local health services or doctors who speak their language [58]. Discrimination by healthcare personnel further exacerbates disparities in vaccination access. Persistent racism contributes to a shortage of health resources for Indigenous communities [48, 57]. While international comparisons, such as those with Australia, highlight shared challenges among Indigenous populations globally, it is crucial to consider Mexico's unique context. The specific dialects, cultural practices, and historical relationships between Indigenous communities and the Mexican healthcare system significantly shape their healthcare experiences. Our survey revealed that 5.6% of respondents' relatives lacked an official identity document, despite all students needing a birth certificate for school enrollment. Nationally, the absence of birth certificates is significantly higher among the Indigenous population (22.75%) compared to the general population (2.1%), particularly among those aged 18 to 59 years (43.6%) and approximately 11,000 older adults. In Mexico, 22.7% of individuals without a birth certificate are Indigenous, with Chiapas ranking first among states, where 23.2% of Indigenous individuals lack this documentation. High levels of marginalization correlate with the highest number of unregistered births [59, 60]. Historically, many older adults prioritized baptismal records over civil birth certificates [61]. However, the prohibitive cost of the civil registry process remains a significant barrier for those in poverty [62]. Sanders and colleagues note this issue is more pronounced in low-income countries with inadequate civil registration systems [63]. The lack of official documentation severely limits access to essential services and fundamental rights [63].

The observed trends in weight gain among participants align with the ENSANUT 2022 findings, which reported national overweight and obesity prevalences of 38.3% and 36.9%, respectively, ranking among the highest globally [64]. The increase in BMI among participants mirrors broader trends during the COVID-19 pandemic, where lifestyle changes led to weight gain in many populations [65]. However, these findings are not central to this study's primary focus, which is the pandemic's impact on COVID-19 infection rates, mortality, and vaccination in a marginalized Indigenous population.

The choice to focus on high school students as the survey population allowed us to gather data efficiently and ensured a higher likelihood of accurate recall and reporting, given their direct involvement in family matters during the pandemic. Due to their unique position within their families, high school students often play a crucial role in translating and conveying health information, especially in communities where language barriers and lower literacy rates among older generations are prevalent. While we acknowledge the inherent limitations in collecting data from this demographic, we believe their perspectives offer crucial insights into the community’s overall experience with COVID-19. This approach provided a reliable source of information. It enabled us to reach a segment of the population often underrepresented in public health research, thereby enriching the study’s findings with diverse viewpoints.

Our findings indicate significant underreporting of the true COVID-19 burden in Indigenous communities. Triangulating survey data with official records clarified the impact of the pandemic. However, several limitations must be noted, including the potential for recall bias, language barriers, and double-counting when reporting COVID-19 deaths among survey participants. It is possible that multiple individuals reported the same death, leading to an inflated count of mortality cases**.** These limitations emphasize the need for more comprehensive data collection methods and targeted interventions to address the challenges faced by Indigenous communities during the pandemic.

The discrepancy between self-reported COVID-19 cases in our survey and official data underscores the broader challenges of accurately capturing the pandemic's impact in marginalized communities like San Juan Chamula. This underreporting is likely due to limited access to testing and healthcare services and a preference for traditional medicine over formal healthcare. These findings highlight the critical need for more robust data collection methods and public health strategies that address the specific needs of Indigenous populations.

## Conclusion

Our study reveals that the absence of ethnic data in national registries leads to a significant underestimation of mortality differentials among Indigenous populations. Despite 14% of our respondents reporting a COVID-19 infection, official records in Chamula documented only 212 cases and one death, highlighting a profound gap in data accuracy**.** Furthermore, with 79.4% of respondents unvaccinated and a considerable portion lacking official birth certificates, our findings point to deep-rooted social exclusion within these communities. These disparities suggest the need for public health measures that more accurately capture and address the unique challenges faced by Indigenous populations.

Tailoring public health strategies to the specific needs of Indigenous populations is essential. This includes integrating local Indigenous languages, cultures, and knowledge systems into public health efforts, with the guidance and leadership of trusted Indigenous leaders. Our findings can inform national strategic plans for pandemic preparedness and response within Indigenous communities.

A key takeaway from our study is the critical importance of investing in education in Indigenous languages to improve adherence to vaccination and other health initiatives. Indigenous peoples have historically been among the most vulnerable during crises, diseases, and pandemics. Prioritizing them in public health care and ensuring interventions are culturally and linguistically appropriate is crucial for protecting and promoting the health of Indigenous communities.

## Supplementary Information

Supplementary Material 1. 

## Data Availability

The datasets used and/or analyzed during the current study are available from the corresponding author on reasonable request.

## Abbreviations

COVID-19: Coronavirus disease
CONAPO: National Population Council
CONEVAL: National Council for the Evaluation of Social Development Policy
IMSS: Instituto Mexicano del Seguro Social
SSA: Secretaria de Salud
WHO: World Health Organization
BMI: Body Mass Index
SPSS: Statistical Package for the Social Sciences
INEGI: National Institute of Statistics and Geography
ENSANUT: National Health and Nutrition Survey

## References

[1] Dahal S, Mamelund SE, Luo R, Sattenspiel L, Self-Brown S, Chowell G. Investigating COVID-19 transmission and mortality differences between indigenous and non-indigenous populations in Mexico. Int J Infect Dis. 2022;122:910–20. 35905949 10.1016/j.ijid.2022.07.052 PMC9357430

[2] INEGI. Información por entidad: Chiapas. Cuéntame: Chiapas. 2020. https://cuentame.inegi.org.mx/monografias/informacion/chis/default.aspx?tema=me&e=07. Accessed 23 Feb 2024.

[3] Consejo Nacional de Evaluación de la Política de Desarrollo Social. Informe de pobreza y evaluación 2022: Chiapas. 1st ed. Ciudad de México: CONEVAL; 2022.

[4] Little BB, Shakib S, Reyes MEP, Karimi S, Vu GT, Dupré N, et al. COVID-19 infection and mortality among non-pregnant indigenous adults in Mexico 2020–2022: Impact of marginalisation. J Glob Health. 2023;13:06030. 37506193 10.7189/jogh.13.06030 PMC10386760

[5] Bayona Escat E. Rituales indígenas y otras escenificaciones turísticas en Los Altos de Chiapas. Nueva Antropología. 2015;XXVIII:31–50.

[6] Consejo Nacional de Población (CONAPO). Índices de marginación 2020. Índices de marginación 2020. 2021. https://www.gob.mx/conapo/documentos/indices-de-marginacion-2020-284372. Accessed 29 Mar 2023.

[7] Cortés F. Consideraciones sobre la marginalidad, marginación, pobreza y desigualdad en la distribución del ingreso. Papeles Poblac. 2002;8:9–24.

[8] Gobierno del Estado de Chiapas. REGIÓN V – ALTOS TSOTSIL TSELTAL. REGIÓN V – ALTOS TSOTSIL TSELTAL. 2013. https://www.ceieg.chiapas.gob.mx/productos/files/MAPASTEMREG/REGION_V_ALTOS_TSOTSIL_TSELTAL_post.pdf. Accessed 10 Oct 2023.

[9] INEGI. Municipios del Estado de Chiapas. Mapa de Chiapas: División Municipal. 2018. https://cuentame.inegi.org.mx/mapas/pdf/entidades/div_municipal/chismpios.pdf. Accessed 26 Nov 2023.

[10] Instituto Nacional de Estadística y Geografía (INEGI). Cuéntame: Chiapas. Cuéntame (Chiapas). 2020. https://cuentame.inegi.org.mx/monografias/informacion/Chis/Poblacion/default.aspx?tema=ME&e=07. Accessed 10 Oct 2023.

[11] Gobierno del Estado de Chiapas. Programa regional de desarrollo: Region V Altos Tsotsil-Tseltal. 2013. https://www.haciendachiapas.gob.mx/planeacion/informacion/desarrollo-regional/prog-regionales/altos.pdf. Accessed 10 Oct 2023.

[12] Consejo Nacional de Ciencia y Tecnología, Gobierno de México. Datos generales sobre COVID-19 en México. Covid-19 México. 2023. https://datos.covid-19.conacyt.mx/#DOView. Accessed 29 Nov 2023.

[13] Sánchez-Pérez HJ, Gordillo-Marroquín C, Vázquez-Marcelín J, Martín-Mateo M, Gómez-Velasco A. Sociodemographic factors associated with the success or failure of anti-tuberculosis treatment in the Chiapas Highlands, Mexico, 2019–2022. PLoS One. 2024;19:e0296924. 38277365 10.1371/journal.pone.0296924 PMC10817218

[14] Coporo Quintana G, Villafuerte SD. Chamula: a migrant town in Los Altos. Chiapas Migración y Desarrollo. 2017;15:97–121.

[15] Page Pliego JT. Aspectos socioculturales que delimitan las diferencias entre los sistemas etnomédicos de Chamula, Chenalhó y Oxchuc en el estado de Chiapas. Revista Pueblos y fronteras digital. 2011;6:123–50.

[16] Rus J. Covid-19 en Chiapas indígena: cuestionando una pandemia oculta. Open Anthropology Research Repository. 2020. https://openanthroresearch.org/index.php/oarr/preprint/view/77/126. Accessed 8 Oct 2023.

[17] Gobierno de México. Municipio de Chamula. Chamula. 2022. https://www.economia.gob.mx/datamexico/es/profile/geo/chamula?healthAreas=ruralHealth&healthIndicators=unitHealth. Accessed 11 Oct 2023.

[18] World Health Organization. WHO Director-General’s opening remarks at the media briefing on COVID-19 - 11 March 2020. WHO Director-General’s opening remarks at the media briefing on COVID-19 - 11 March 2020. 2020. https://www.who.int/director-general/speeches/detail/who-director-general-s-opening-remarks-at-the-media-briefing-on-covid-19---11-march-2020. Accessed 10 Oct 2023.

[19] Sol de México. Indígenas de Chiapas desafían cuarentena con procesión de Semana Santa. Indígenas de Chiapas desafían cuarentena con procesión de Semana Santa. 2020.

[20] Cámara de Diputados, Secretaría de Educación Pública, Secretaría de Gobernación. ACUERDO número 02/03/20 por el que se suspenden las clases en las escuelas de educación preescolar, primaria, secundaria, normal y demás para la formación de maestros de educación básica del Sistema Educativo Nacional, así como aquellas de los tipos medio superior y superior dependientes de la Secretaría de Educación Pública. Ciudad de México; 2022.

[21] Suárez V, Suarez Quezada M, Oros Ruiz S, De RonquilloJesús E. Epidemiology of COVID-19 in Mexico: from the 27th of February to the 30th of April 2020. Rev Clin Esp. 2020;220:463–71. 33994571 10.1016/j.rce.2020.05.007 PMC7250750

[22] Gobierno de México. Datos COVID-19 en México. Datos COVID-19 en México. 2023. https://datos.covid-19.conacyt.mx/#COMNac. Accessed 11 Oct 2023.

[23] Gobierno de México. Covid-19 México-Mapa Municipal. Covid-19 México-Mapa Municipal. https://datos.covid-19.conacyt.mx/fHDMap/mun.php. Accessed 11 Oct 2023.

[24] Diaz E, Dimka J, Mamelund S-EM. Disparities in the offer of COVID-19 vaccination to migrants and non-migrants in Norway: a cross sectional survey study. BMC Public Health. 2022;22:1–12. 10.1186/s12889-022-13687-8 PMC9252073 35788219

[25] Joaquin Damas E, Dahal S, Rivera Aguilar AG, Garcia Morales J, Sattenspiel L, Mamelund SE, et al. Attitudes and behaviors of university students during the COVID-19 pandemic in a predominantly Indigenous population in Mexico: a survey study. Discov Soc Sci Health. 2023;3:16.

[26] Fernández Ham P. Indígenas de sociedades contemporáneas: retos estadísticos. 2010:1–27. https://www.inegi.org.mx/contenidos/eventos/2010/genero/01_Patricia%20FernandezHam_IndigenasAgs2010.pdf. Accessed 29 June 2022.

[27] Cámara de Diputados. La definición de indígena en el ámbito internacional. 2003. http://www.diputados.gob.mx/bibliot/publica/inveyana/polisoc/derindi/3ladefin.htm. Accessed 28 Aug 2022.

[28] World Medical Association. World Medical Association Declaration of Helsinki. WMA Declaration of Helsinki – Ethical Principles for Medical Research Involving Human Subjects. 2013. https://www.wma.net/policies-post/wma-declaration-of-helsinki-ethical-principles-for-medical-research-involving-human-subjects/. Accessed 8 Aug 2023.

[29] Gobierno de Mexico. Incremento a los Salarios Mínimos para 2024. 2023. https://www.gob.mx/conasami/articulos/incremento-a-los-salarios-minimos-para-2024?idiom=es. Accessed 29 Aug 2024.

[30] CHOPO laboratorios. Detección COVID-19 por PCR. Detección de SARS COV-2 RNA (COVID-19) por PT-PCR. 2022. https://www.chopo.com.mx/metro/deteccion-covid-19-por-pcr. Accessed 6 Sep 2022.

[31] Palacio-Mejía LS, Hernández-Ávila JE, Hernández-Ávila M, Dyer-Leal D, Barranco A, Quezada-Sánchez AD, et al. Leading causes of excess mortality in Mexico during the COVID-19 pandemic 2020–2021: a death certificates study in a middle-income country. Lancet Reg Health Am. 2022;13:1–15. 10.1016/j.lana.2022.100303 PMC9230439 35782204

[32] Instituto Nacional de los Pueblos Indígenas (INPI). Vacunación en México. El INPI implementa acciones de información, prevención y mitigación en pueblos indígenas ante el COVID-19. 2020. https://www.gob.mx/inpi/articulos/el-inpi-implementa-acciones-en-pueblos-y-comunidades-indigenas-y-afromexicanas-ante-el-covid-19. Accessed 23 May 2024.

[33] Richardson L, Crawford A. COVID-19 and the decolonization of Indigenous public health. CMAJ. 2020;192:E1098–100. 32958575 10.1503/cmaj.200852 PMC7532009

[34] Takasaki Y, Abizaid C, Coomes OT. COVID-19 contagion across remote communities in tropical forests. Sci Rep. 2022;12:20727. 36456613 10.1038/s41598-022-25238-7 PMC9713114

[35] Gomes Machado FC, Ferron MM, Da TerezaMattaBarddal M, Nascimento LA, Rosalen J, Avelino-Silva VI. COVID-19 vaccination, incidence, and mortality rates among indigenous populations compared to the general population in Brazil: Describing trends over time. Lancet Reg Health Am. 2022;13:100319. 35874497 10.1016/j.lana.2022.100319 PMC9294659

[36] Cortés-Meda A, Ponciano-Rodríguez P. Impacto de los determinantes sociales de la COVID-19 en México. Boletín sobre COVID-19. 2021;2:9–13.

[37] Soares GH, Jamieson L, Biazevic MGH, Michel-Crosato E. Disparities in Excess Mortality Between Indigenous and Non-Indigenous Brazilians in 2020: Measuring the Effects of the COVID-19 Pandemic. J Racial Ethn Health Disparities. 2022;9:2227–36. 34581998 10.1007/s40615-021-01162-w PMC8477716

[38] Buszkiewicz JH, Hajat A, Hill HD, Otten JJ, Drewnowski A. Racial, ethnic, and gender differences in the association between higher state minimum wages and health and mental well-being in US adults with low educational attainment. Soc Sci Med. 2023;322:115817. 36905725 10.1016/j.socscimed.2023.115817 PMC11321499

[39] Muñoz Ramírez G. Los de abajo. La Jornada: Opinión; 2020. https://www.jornada.com.mx/2020/05/02/opinion/017o1pol. Accessed 11 Feb 2024.

[40] Petrov AN, Welford M, Golosov N, DeGroote J, Devlin M, Degai T, et al. Lessons on COVID-19 from Indigenous and remote communities of the Arctic. Nat Med. 2021;27:1491–2. 34426705 10.1038/s41591-021-01473-9

[41] Villela S. Indigenous Peoples and covid-19 Syndemic in Mexico. ANTROPOLOGÍA: Revista Interdisciplinaria del INAH. 2020;4:124–40.

[42] Banning J. How Indigenous people are coping with COVID-19. CMAJ. 2020;192:E787–8. 32631914 10.1503/cmaj.1095879 PMC7828894

[43] Viscogliosi C, Asselin H, Basile S, Borwick K, Couturier Y, Drolet M-J, et al. Importance of Indigenous elders’ contributions to individual and community wellness: results from a scoping review on social participation and intergenerational solidarity. Can J Public Health. 2020;111:667–81. 32109314 10.17269/s41997-019-00292-3 PMC7501322

[44] Serván-Mori E, Seiglie JA, Gómez-Dantés O, Wirtz VJ. Hospitalisation and mortality from COVID-19 in Mexican indigenous people: A cross-sectional observational study. J Epidemiol Community Health. 1978;2022(76):16–23. 10.1136/jech-2020-216129 34266980

[45] WHO. Diabetes. Diabetes. 2024. https://www.who.int/health-topics/diabetes#tab=tab_1. Accessed 11 Feb 2024.

[46] Contreras Manzano A, Guerrero López CM, Aguerrebere M, Sedas C, Lamadrid FH. Municipality-Level Predictors of COVID-19 Mortality in Mexico: a cautionary tale. Disaster Med Public Health Prep. 2022;16:1384–92. 33731243 10.1017/dmp.2020.485 PMC7985638

[47] Chiquete E, Alegre-Díaz J, Ochoa-Guzmán A, Toapanta-Yanchapaxi LN, González-Carballo C, Garcilazo-Ávila A, et al. Ethnicity and other COVID-19 death risk factors in Mexico. Arch Med Sci. 2022;18:711–8. 35591829 10.5114/aoms.2020.101443 PMC9103400

[48] Taikeff N, Achkar A, Naous E, Mitri J. Unspoken consequences of structural racism in the USA: diabetes and COVID-19. J Racial Ethn Health Disparities. 2023;11:2575–82. 37460920 10.1007/s40615-023-01722-2

[49] Guarchaj M, Tschida S, Milian Chew JP, Aguilar A, Flood D, Fort MP, et al. Impact of COVID-19 on diabetes care: mixed methods study in an Indigenous area of Guatemala. BMJ Open. 2024;14:e079130. 38167279 10.1136/bmjopen-2023-079130 PMC10773399

[50] de DíazLeón-Martínez L, de la Sierra-de la Vega L, Palacios-Ramírez A, Rodriguez-Aguilar M, Flores-Ramírez R. Critical review of social, environmental and health risk factors in the Mexican indigenous population and their capacity to respond to the COVID-19. Sci Total Environ. 2020;733:139357. 32416536 10.1016/j.scitotenv.2020.139357 PMC7215151

[51] Robledo AZ. La Encuesta Nacional de Salud y Nutrición 2022. Salud Publica Mex. 2023;65:S1–4. 38060939 10.21149/15087

[52] World Health Organization. COVID-19 Mexico situation . Vaccination in Mexico. 2023. https://data.who.int/dashboards/covid19/vaccines?n=c. Accessed 23 May 2024.

[53] Hernández-Puente D, Cruz-Martínez D, Luna-Ávila S. COVID-19: Inmunizaciones en México, marzo 2021. Boletín sobre COVID-19 - Salud Pública y Epidemiología. 2021;2:19–23.

[54] Mansilla Corona R. Teorías de la conspiración, fake news y COVID-19. In: Centro de Investigaciones Interdisciplinarias en Ciencias y Humanidades, Consejo Mexicano de Ciencias Sociales AC, editors. Las ciencias sociales y el coronavirus. 1st edition. Mexico: Libros UNAM; 2022. p. 59–74.

[55] Barbosa MDS, Croda MG, Simionatto S. Vaccination against covid-19 in the brazilian indigenous population: Has science been defeated by fake news? Rev Soc Bras Med Trop. 2021;54:e02722021. 34431945 10.1590/0037-8682-0272-2021 PMC8405215

[56] UNESCO. El impacto de la desinformación en las poblaciones indígenas. 1st edition. París, France: UNESCO; 2022.

[57] Aylsworth L, Manca T, Dubé È, Labbé F, Driedger SM, Benzies K, et al. A qualitative investigation of facilitators and barriers to accessing COVID-19 vaccines among Racialized and Indigenous Peoples in Canada. Hum Vaccin Immunother. 2022;18:2129827. 36218335 10.1080/21645515.2022.2129827 PMC9746392

[58] Graham S, Blaxland M, Bolt R, Beadman M, Gardner K, Martin K, et al. Aboriginal peoples’ perspectives about COVID-19 vaccines and motivations to seek vaccination: a qualitative study. BMJ Glob Health. 2022;7:e008815. 35858705 10.1136/bmjgh-2022-008815 PMC9304971

[59] Prado Solé E, Mier Bueno L, Álvarez Gutiérrez M, Andrade Zamorano N, Gaytán Nieto R, Martínez Herrera MÁ. Situación actual del registro de nacimiento en el país. In: Derecho a la identidad. La cobertura del registro de nacimiento en México. Mexico: UNICEF, INEGI; 2018. p. 18–22.

[60] Instituto Nacional de Estadística y Geografía. La cobertura del registro de nacimiento en México”. SEIS DE CADA 10 PERSONAS SIN REGISTRO EN EL PAÍS SON UN NIÑO, NIÑA O ADOLESCENTE. 2019. https://www.inegi.org.mx/contenidos/saladeprensa/boletines/2019/EstSociodemo/identidad2019.pdf. Accessed 28 Apr 2024.

[61] Rosales Ramirez I. Mexicans without identity. Gaceta Cusur. 2020. http://gaceta.cusur.udg.mx/mexicanos-sin-identidad/. Accessed 16 Feb 2024.

[62] Gonzales GS. Un millón de mexicanos, sin acta de nacimiento ni plenos derechos. Periódico La Jornada. 2019. https://www.jornada.com.mx/2019/01/23/sociedad/031n1soc. Accessed 16 Feb 2024.

[63] Sanders C, Burnett K. A case study in personal identification and social determinants of health: Unregistered births among indigenous people in Northern Ontario. Int J Environ Res Public Health. 2019;16:567. 30781459 10.3390/ijerph16040567 PMC6406902

[64] Campos-Nonato I, Galván-Valencia O, Hernández-Barrera L, Oviedo-Solís C, Barquera S. Prevalence of obesity and associated risk factors in Mexican adults: results of the Ensanut 2022. Salud Publica Mex. 2023;65:s238–47. 38060949 10.21149/14809

[65] Shamah-Levy T, Gaona-Pineda EB, Cuevas-Nasu L, Morales-Ruan C, Valenzuela-Bravo DG, Humarán IMG, et al. Prevalence of overweight and obesity in Mexican school-aged children and adolescents. Ensanut 2020–2022. Salud Publica Mex. 2023;65:4–10.

