# Supplementary material for: Evaluating COVID-19 impact, vaccination, birth registration, and underreporting in a predominantly indigenous population in Chiapas, Mexico
Source: BMC Infect Dis. 2024 Dec 3;24:1376. doi: 10.1186/s12879-024-10156-y (PMC11613722; doi:10.1186/s12879-024-10156-y)
Supplement: Supplementary file 1 — Supplementary Material 1. [file 12879_2024_10156_MOESM1_ESM.docx]

**Appendix**

Appendix 1. CONAPO indicators for the measurement of the marginalization index

| Concept | Socioeconomic dimensions | Exclusion data | Indicator to measure the intensity of exclusion | Marginalization index |
| --- | --- | --- | --- | --- |
| Multiple structural phenomenon that assesses dimensions, forms and intensities of exclusion in the process of development and enjoyment of its benefits. | Education | Illiteracy | Percentage of population 15 years of age and over illiterate | Overall intensity of socioeconomic marginalization |
|  |  | Population without  complete basic education | Percentage of population 15 years old and over without complete basic education |  |
|  | Housing | Private homes without  drainage or sanitation | Percentage of occupants in private dwellings without drainage or sanitation |  |
|  |  | Private homes without  electricity | Percentage of occupants in private dwellings without electricity |  |
|  |  | Private homes without  piped water | Percentage of occupants in private housing without piped water |  |
|  |  | Private homes with  dirt floors | Percentage of occupants in private dwellings with dirt floor |  |
|  |  | Overcrowded private  housing | Percentage of private dwellings with overcrowding |  |
|  | Population distribution | Localities with less  than 5,000 inhabitants | Percentage of population residing in localities with less than 5,000 inhabitants |  |
|  | Monetary income | Employed population  earning up to two  minimum wages | Percentage of employed population with incomes up to two minimum wages |  |

Appendix 2. Chiapas Municipalities with a Very High Marginalization Degree (Part 1/2)

| Municipality | Total population 2020 | % Illiterate population 15 years of age and older | % Population 15 years and older without basic education | % Occupants in private dwellings without drainage or toilet | % Occupants in private homes without electricity | % Occupants in private dwellings without piped water | % Occupants in private dwellings with dirt floors | % Overcrowded private housing | % Population in towns with less than 5,000 inhabitants | % Employed population with income <2 minimum wages | Marginalization Index 2020 | Degree of Marginalization 2020 | Deaths due COVID-19 to 06/23/2023 |
| --- | --- | --- | --- | --- | --- | --- | --- | --- | --- | --- | --- | --- | --- |
| Amatenango del Valle | 11 283 | 26.49 | 75.01 | 12.81 | 1.54 | 27.52 | 19.34 | 49.04 | 47.53 | 97.19 | 45.982 | Very High | 0 |
| Chalchihuitán | 21 915 | 29.17 | 81.16 | 15.48 | 11.56 | 48.64 | 39.07 | 63.17 | 100.00 | 98.30 | 37.807 | Very High | 0 |
| Chamula | 101 967 | 32.59 | 86.14 | 1.85 | 0.85 | 17.77 | 18.69 | 49.70 | 100.00 | 98.61 | 46.328 | Very High | 1 |
| Chanal | 13 678 | 22.38 | 59.34 | 2.89 | 2.58 | 34.39 | 25.97 | 63.20 | 30.30 | 97.78 | 46.438 | Very High | 0 |
| Chenalhó | 47 371 | 27.61 | 80.15 | 3.63 | 2.55 | 18.87 | 32.96 | 50.77 | 100.00 | 97.94 | 45.429 | Very High | 0 |
| Chilón | 137 262 | 26.65 | 60.49 | 10.54 | 6.06 | 13.68 | 24.76 | 57.23 | 88.59 | 96.06 | 45.256 | Very High | 2 |
| Huixtán | 22 975 | 18.27 | 63.15 | 0.74 | 2.87 | 22.46 | 20.08 | 39.49 | 100.00 | 96.03 | 48.532 | Very High | 2 |
| Mitontic | 13 755 | 36.64 | 79.48 | 1.36 | 0.57 | 24.50 | 25.41 | 41.48 | 100.00 | 96.62 | 45.571 | Very High | 0 |
| Ocosingo | 234 661 | 21.92 | 56.95 | 5.18 | 6.86 | 11.68 | 29.42 | 49.24 | 69.76 | 93.26 | 47.064 | Very High | 29 |
| Oxchuc | 54 932 | 15.99 | 52.87 | 4.34 | 10.44 | 70.05 | 41.20 | 53.73 | 81.15 | 95.99 | 40.834 | Very High | 0 |

Appendix 3. Chiapas Municipalities with a Very High Marginalization Degree (Part 2/2)

| Municipality | Total population 2020 | % Illiterate population 15 years of age and older | % Population 15 years and older without basic education | % Occupants in private dwellings without drainage or toilet | % Occupants in private homes without electricity | % Occupants in private dwellings without piped water | % Occupants in private dwellings with dirt floors | % Overcrowded private housing | % Population in towns with less than 5,000 inhabitants | % Employed population with income <2 minimum wages | Marginalization Index 2020 | Degree of Marginalization 2020 | Deaths due COVID-19 to 06/23/2023 |
| --- | --- | --- | --- | --- | --- | --- | --- | --- | --- | --- | --- | --- | --- |
| Pantelhó | 26 391 | 36.02 | 74.84 | 6.49 | 2.98 | 10.79 | 21.82 | 60.90 | 67.30 | 97.53 | 45.494 | Very High | 0 |
| Salto de Agua | 64 251 | 23.15 | 55.22 | 8.25 | 3.56 | 8.33 | 17.44 | 50.25 | 90.38 | 94.74 | 48.093 | Very High | 4 |
| Simojovel | 52 935 | 24.61 | 62.85 | 2.27 | 1.00 | 7.32 | 20.18 | 63.51 | 72.39 | 93.77 | 48.699 | Very High | 2 |
| Sitalá | 15 518 | 40.11 | 73.88 | 45.44 | 7.06 | 39.77 | 32.22 | 52.72 | 100.00 | 97.42 | 35.902 | Very High | 0 |
| Tila | 83 505 | 23.04 | 56.47 | 0.84 | 3.99 | 9.22 | 28.67 | 47.35 | 79.61 | 95.18 | 48.340 | Very High | 9 |
| Tumbalá | 38 025 | 26.70 | 57.50 | 4.83 | 3.72 | 18.88 | 11.64 | 45.84 | 100.00 | 94.36 | 47.478 | Very High | 1 |
| Zinacantán | 45 373 | 31.02 | 84.08 | 3.57 | 0.44 | 26.31 | 12.12 | 46.25 | 64.68 | 98.63 | 46.922 | Very High | 1 |
| San Juan Cancuc | 37 948 | 30.80 | 69.80 | 9.42 | 2.75 | 27.05 | 30.22 | 55.85 | 77.66 | 98.85 | 44.109 | Very High | 0 |
| Maravilla Tenejapa | 14 714 | 19.21 | 69.42 | 2.41 | 3.17 | 16.68 | 13.30 | 50.22 | 100.00 | 88.14 | 48.672 | Very High | 0 |
| Santiago el Pinar | 4 959 | 33.21 | 62.62 | 2.06 | 0.50 | 1.03 | 21.66 | 53.68 | 100.00 | 99.22 | 47.998 | Very High | 0 |

Appendix 4. Chiapas Municipalities with a Low Marginalization Degree

| Municipality | Total population 2020 | % Illiterate population 15 years of age and older | % Population 15 years and older without basic education | % Occupants in private dwellings without drainage or toilet | % Occupants in private homes without electricity | % Occupants in private dwellings without piped water | % Occupants in private dwellings with dirt floors | % Overcrowded private housing | % Population in towns with less than 5,000 inhabitants | % Employed population with income <2 minimum wages | Marginalization Index 2020 | Degree of Marginalization 2020 | Deaths due COVID-19 to 06/23/2023 |
| --- | --- | --- | --- | --- | --- | --- | --- | --- | --- | --- | --- | --- | --- |
| Comitán de Domínguez | 166 178 | 9.11 | 44.35 | 0.56 | 0.54 | 13.03 | 6.48 | 23.32 | 31.71 | 82.53 | 54.968 | Low | 38 |
| Chiapa de Corzo | 112 075 | 9.11 | 37.21 | 1.02 | 0.63 | 7.11 | 6.11 | 33.03 | 42.96 | 80.87 | 55.037 | Low | 60 |
| Reforma | 44 829 | 6.54 | 35.69 | 1.09 | 0.37 | 3.57 | 5.61 | 31.67 | 35.27 | 73.36 | 56.284 | Low | 52 |
| San Cristóbal de las Casas | 215 874 | 9.22 | 38.40 | 0.55 | 0.43 | 6.55 | 10.42 | 25.15 | 14.99 | 77.11 | 55.833 | Low | 77 |
| Tapachula | 353 706 | 6.89 | 34.11 | 0.92 | 0.74 | 8.14 | 10.37 | 28.43 | 31.93 | 78.63 | 55.512 | Low | 518 |
| Tapilula | 13 592 | 11.63 | 34.76 | 0.42 | 0.56 | 2.12 | 9.86 | 38.04 | 38.04 | 87.77 | 54.631 | Low | 4 |
| Tonalá | 91 913 | 9.36 | 40.03 | 1.69 | 0.67 | 6.95 | 3.47 | 29.79 | 51.18 | 80.08 | 55.072 | Low | 72 |

Survey in Spanish

| 1.Edad |
| --- |
| 2.¿Cual es tu genero?   1. Hombre 2. Mujer |
| 3.Talla/Estatura (en centímetros) |
| 4.¿Cuál es tu peso actual en kilogramos? |
| 5.¿Cuál era tu peso al incio de la pandemia? (Enero 2020) |
| 6.¿Hablas algún dialecto?   1. Si 2. No |
| 7.¿Cuál?  a) Tseltal (winik atel)  b) Tsotsi (batsil winik' otik)  c) Ch'ol (winik)  d) Zoque (o' de put)  e) Tojolabal (tojolwinik'otik)  f) Mam (mam)  g) Mochó (mochó o motozintleco)  h) Kaqchikel (kaqchiquel)  i) Jakalteco (jakalteko)  j) Chuj (chuj)  k) Q'anjobal (q'anjobal)  l) Lacandón (hach winik)  m) No hablo ninguno |
| 8.¿Tu familia habla algún dialecto?   1. Si 2. No |
| 9.¿Cuál?  a) Tseltal (winik atel)  b) Tsotsi (batsil winik' otik)  c) Ch'ol (winik)  d) Zoque (o' de put)  e) Tojolabal (tojolwinik'otik)  f) Mam (mam)  g) Mochó (mochó o motozintleco)  h) Kaqchikel (kaqchiquel)  i) Jakalteco (jakalteko)  j) Chuj (chuj)  k) Q'anjobal (q'anjobal)  l) Lacandón (hach winik)  m) No hablo ninguno |
| 10.¿Te consideras a ti mismo como perteneciente a un grupo indígena?   1. Si 2. No |
| 11. ¿Con cuantas personas viven en tu casa?   1. 1 a 3 2. 4 a 6 3. 7 a 10 4. Mas de 10 |
| 12.¿Te has enfermado de COVID-19?  a) Sí  b) No |
| 13.¿Cuántas veces te has enfermado de COVID-19?   1. 0 2. 1 3. 2 4. 3 5. 4 6. 5 7. Más de 5 |
| 14.¿Qué tratamiento tuviste cuando te enfermaste de COVID-19?   1. Medicina tradicional de mi comunidad (rituales, chamán, curandero) 2. Medicina en un hospital público 3. Medicina en un hospital privado 4. Medicina en alguna farmacia del Dr Simi, Farmacia del Ahorro o equivalente 5. Ninguna de las anteriores 6. No me he enfermado |
| 15.¿Alguien de los que viven en tu casa se han enfermado de COVID-19?   1. Si 2. No |
| 16.¿Quién en tu casa se ha enfermado de COVID-19?   1. Padres 2. Hermanos mayores de 18 años 3. Hermanos menores de 18 años 4. Abuelos 5. Nadie 6. Otros |
| 17.¿Alguien en tu casa fue o ha sido hospitalizado con COVID-19?   1. Si 2. No |
| 18.¿Quien en tu casa fue o ha sido hospitalizado por COVID-19?   1. Padres 2. Hermanos mayores de 18 años 3. Hermanos mennores de 18 años 4. Abuelos 5. Nadie 6. Otro |
| 19.¿Alguien en tu casa murió por COVID-19?   1. Si 2. No |
| 20.¿Quien en tu casa murió por COVID-19?   1. Padres 2. Hermanos mayores de 18 años 3. Hermanos menores de 18 años 4. Abuelos 5. Nadie 6. Otros |
| 21.¿Sabes cuántas muertes aproximadamente hubo en tu paraje/comunidad por causa de COVID-19?  a) Ninguno  b) 1 a 5  c) 6 a 10  d 11 a 15  e)16 a 20  f)Más de 20  g)No sé |
| 22.¿Sabes si las muertes por causa de COVID-10 de tus familiares fueron registradas?   1. Si, en el comité de mi paraje, 2. Si, en el registro de la iglesia de mi paraje 3. Si, en la presidencia municipal 4. No fueron registradas 5. No sé |
| 23.¿Alguien más en tu paraje ha muerto por algo que NO tiene que ver con COVID-19 durante la pandemia? (Enero-2020 a Mayo 2023)   1. Si, diabetes 2. Si, presion alta 3. Si, enfermedad renal 4. Si, enfermedad del corazón 5. Si, grasa alta 6. Si, obesidad 7. Si, tumores/cancer 8. Si, agresiones 9. Si, influenza/pulmonía 10. No, nadie murió por algo que NO tiene que ver con COVID-19 11. No sé |
| 24.¿Te han ofrecido una vacuna contra COVID-19?   1. Si 2. No |
| 25.¿Has sido vacunado contra COVID-19?   1. Si 2. No |
| 26.¿Cuántas dosis de vacunas contra COVID-19 tienes?  a) 0  b) 1  c) 2  d) 3  e) 4  f) 5  g) Más de 5 |
| 27.¿Dónde fuiste vacunado contra COVID-19?   1. En la clínica de mi paraje 2. En campaña de vacunación en mi paraje 3. En campaña de vacunación en mi escuela 4. Tuve que ir a la ciudad más cercana 5. No he sido vacunado |
| 28.¿Te explicaron como funciona la vacuna en tu dialecto natal?   1. Si 2. No 3. No, solo en español 4. No, no me explicaron |
| 29.¿Quien más en tu casa ha sido vacunado contra COVID-19?   1. Padres 2. Hermanos mayores de 18 años 3. Hermanos menores de 18 años 4. Abuelos 5. Otros 6. Mi familia no se ha vacunado |
| 30.¿Le explicaron a tu familia como funciona la vacuna en tu dialecto natal?   1. Si 2. No 3. No, solo español 4. No, no les explicaron 5. Mi familia no se ha vacunado |
| 31.¿Todos en tu casa cuentan con un acta de nacimiento oficial (obtenido de la presidencia municipal)?   1. Si 2. No |
| 32.¿Quién en tu casa NO cuenta con acta de nacimiento oficial (obtenido de la presidnecia municipal)?   1. Padres 2. Hermanos mayores de 18 años 3. Hermanos menores de 18 años 4. Abuelos 5. Otro integrante 6. Todos tenemos acta de nacimiento oficial |
| 33.¿Conoces a alguien fuera de tu familia que no cuente con un acta de nacimiento?   1. Si 2. No 3. No sé |

Survey in English

| 1.Age |
| --- |
| 2.What is your gender?  a) Male  b) Female |
| 3. What is your height? (cm) |
| 4. What is your current weight in kilograms? |
| 5. What was your weight before the pandemic? (January 2020) |
| 6. Do you speak a dialect?  a) Yes  b) No |
| 7. What dialect do you speak?  a) Tseltal (winik atel)  b) Tsotsi (batsil winik' otik)  c) Ch'ol (winik)  d) Zoque (o' de put)  e) Tojolabal (tojolwinik'otik)  f) Mam (mam)  g) Mochó (mochó o motozintleco)  h) Kaqchikel (kaqchiquel)  i) Jakalteco (jakalteko)  j) Chuj (chuj)  k) Q'anjobal (q'anjobal)  l) Lacandón (hach winik)  m) Do not speak a dialect |
| 8. Do you family speak any dialect?  a) Yes  b) No |
| 9. Which dialect do they speak?  a) Tseltal (winik atel)  b) Tsotsi (batsil winik' otik)  c) Ch'ol (winik)  d) Zoque (o' de put)  e) Tojolabal (tojolwinik'otik)  f) Mam (mam)  g) Mochó (mochó o motozintleco)  h) Kaqchikel (kaqchiquel)  i) Jakalteco (jakalteko)  j) Chuj (chuj)  k) Q'anjobal (q'anjobal)  l) Lacandón (hach winik)  m) They do not speak a dialect |
| 10. Do you consider yourself as belonging to an Indigenous group?  a) Yes  b) No |
| 11. How many people live in your house?   1. 1 a 3 2. 4 a 6 3. 7 a 10 4. More than 10 |
| 12. Have you been sick with COVID-19?  a) Yes  b) No |
| 13. How many times have been sick with COVID-19?   1. 0 2. 1 3. 2 4. 3 5. 4 6. 5 7. More than 5 |
| 14. What treatment did you have when you got sick with COVID-19?   1. Traditional medicine of my community (rituals, shaman, healer) 2. Medicine in a public hospital 3. Medicine in a private hospital 4. Medicine at a Dr Simi's pharmacy, Farmacia del Ahorro or equivalent 5. None of the above 6. I have not been sick |
| 15. Has anyone in your household been sick with COVID-19?  a) Yes  b) No |
| 16. Who in your household has been sick with COVID-19?  a) Father  b) Sibling (s) over 18 years of age  c) Sibling (s) under 18 years of age  d) Grandfathers  e) No one  f) Others |
| 17. Has anyone in your household been hospitalized due COVID-19?  a) Yes  b) No |
| 18. Who in your household has been hospitalized due COVID-19?  a) Father  b) Sibling (s) over 18 years of age  c) Sibling (s) under 18 years of age  d) Grandfathers  e) No one  f) Others |
| 19. Has anyone in your household died due COVID-19?  a) Yes  b) No |
| 20. Who in your household has died due COVID-19?  a) Father  b) Sibling (s) over 18 years of age  c) Sibling (s) under 18 years of age  d) Grandfathers  e) No one  f) Others |
| 21. Do you know approximately how many deaths there were in your area/community due to COVID-19?  a) No one  b) 1 a 5  c) 6 a 10  d 11 a 15  e) 16 a 20  f) More than 20  g) Do not know |
| 22. Do you know if the COVID-10 deaths of your family members were registered?  a) Yes, in the committee of the community  b) Yes, in the registry of the church of the community  c) Yes, at the municipality  d) Not registered  e) Do not know |
| 23. Has anyone else in your area died from anything NOT related to COVID-19 during the pandemic (January-2020 to May 2023)?  a) Yes, diabetes  b) Yes, hypertension  c) Yes, heart disease  d) Yes, renal disease  e) Yes, hypercholesterolemia  f) Yes, obesity  g) Yes, tumors/cancer  h) Yes, aggression  i) Yes, influenza/pneumonia  j) No, no one died for anything NOT related to COVID-19.  k) Do not know |
| 24. Have you been offered a vaccine against COVID-19?  a) Yes  b) No |
| 25. Have you been vaccinated against COVID-19?  a) Yes  b) No |
| 26. How many doses of COVID-19 vaccine do you have?  a) 0  b) 1  c) 2  d) 3  e) 4  f) 5  g) More than 5 |
| 27. Where were you vaccinated against COVID-19?  a) At the clinic in the community  b) Vaccination campaign in the community  c) Vaccination campaign at school  d) In the nearest city  e) Without vaccination |
| 28. Did they explain how the vaccine works in your native dialect?  a) Yes  b) No  c) No, only Spanish  d) No, they did not explain |
| 29. Who else in your household has been vaccinated against COVID-19?  a) Father  b) Sibling (s) over 18 years of age  c) Sibling (s) under 18 years of age  d) Grandfathers  e) No one  f) Others |
| 30. Did they explain how the vaccine works in your native dialect?  a) Yes  b) No  c) No, only Spanish  d) No, they did not explain |
| 31. Does everyone in your household have an official birth certificate (obtained from the municipality)?  a) Yes  b) No |
| 32. Who in your household does NOT have an official birth certificate (obtained from the municipal presidency)?  a) Father  b) Sibling (s) over 18 years of age  c) Sibling (s) under 18 years of age  d) Grandfathers  e) No one  f) Others |
| 33. Do you know anyone outside your family who does not have a birth certificate?  a) Yes  b) No  c) Do not know |
